# Supplementary material for: Intersectional disparities in mental healthcare utilization by sex and race/ethnicity among US adults: An NHANES study
Source: PLOS Glob Public Health. 2025 Oct 13;5(10):e0004682. doi: 10.1371/journal.pgph.0004682 (PMC12517490; doi:10.1371/journal.pgph.0004682)

PLOS Global Public Health

Intersectional Disparities in Mental Healthcare Utilization by Sex and Race/Ethnicity among US Adults: An NHANES Study

--Manuscript Draft--

**Manuscript Number:**	PGPH-D-25-01113
**Article Type:**	Research Article
**Full Title:**	Intersectional Disparities in Mental Healthcare Utilization by Sex and Race/Ethnicity among US Adults: An NHANES Study
**Short Title:**	Intersectional Disparities in Mental Healthcare Utilization in the US
**Corresponding Author:**	Lotenna Olisaeloka<br>The University of British Columbia<br>Vancouver, British Columbia CANADA
**Order of Authors:**	Lotenna Olisaeloka, MD, MSc.
	Gentille Musengimana, MSc
	Esteban J. Valencia, MPH
	Daniel Vigo, MD, Dr.PH
	Ehsan Karim, Ph.D
**Keywords:**	Mental Health; Intersectionality; Race/Ethnicity; Sex; Health Disparities; Equity; Socioeconomic Status; Mental Healthcare Access
**Abstract:**	Introduction: Mental healthcare utilization in the US remains low, with sociodemographic factors like sex and race/ethnicity influencing access across different population groups. However, how these factors interact to shape service utilization remains understudied.Methods: Using data from the 2009–2018 cycles of the National Health and Nutrition Examination Survey (NHANES), we employed design-based log-binomial models to estimate the relative differences (prevalence ratios) of mental healthcare utilization across intersecting sex and race/ethnic groups. To assess the absolute differences, we used linear probability regression models to estimate the prevalence differences across these intersectional groups. We also conducted stratified analyses by education, income, health insurance, and depression status to examine whether disparities persisted across socioeconomic and health-related subgroups.Results: Overall, 9.1% of adults reported accessing mental health services in the past year. Hispanic males had the lowest utilization rates compared to Non-Hispanic (NH) White males, with an adjusted prevalence ratio (aPR) of 0.59 [95% CI: 0.47–0.73]. Among females, significant disparities were observed across all race/ethnic minority groups, with NH Black and Hispanic females having significantly lower utilization rates compared to NH White females. Absolute prevalence differences mirrored the relative measures. Stratified analyses were largely consistent with primary results but revealed some variation across education, income, and health insurance strata.Discussion: These findings reflect how intersecting sociodemographic characteristics, specifically sex and race/ethnicity, influence utilization of mental health services. Stratified results suggest that socio-economic status may modify these disparities, pointing to the role played by systemic inequities. Hence, culturally informed strategies and wider structural interventions are needed to address these disparities. Future research should consider additional intersecting identities (e.g., sexual orientation and disability) and investigate socio-structural approaches to reducing these gaps.
**Additional Information:**	
**Question**	**Response**
**Financial Disclosure**	No funding was sought or used for this research.

Powered by Editorial Manager® and ProduXion Manager® from Aries Systems Corporation

Enter a financial disclosure statement that describes the sources of funding for the work included in this submission and the role the funder(s) played. This includes grants and any commercial funding of the work or authors.<br><br>This statement will be typeset if the manuscript is accepted for publication.<br><br>*Please review the submission guidelines and the instructions link below for detailed requirements and guidance.*	
**Competing Interests**<br><br>On behalf of all authors, disclose any competing interests that could be perceived to bias this work.<br><br>This statement will be typeset if the manuscript is accepted for publication.<br><br>*Please review the instructions link below and PLOS Global Public Health's competing interests policy to determine what information must be disclosed at submission.*	Authors declare no conflict of interest.
**Data Availability**<br><br>Before publication, Authors are required to make fully available and without restriction all data underlying their findings. Please see our PLOS Data Policy page for detailed information on this policy.<br><br>A **Data Availability Statement**, detailing where the data can be accessed, is required at first submission. Insert your Data Availability Statement in the box below.	The National Health and Nutrition Survey (NHANES) dataset used for this study is publicly available via the CDC website.

Please see the data reporting section of our submission guidelines for instructions on what you need to include in your Data Availability Statement.<br><br>This statement will be typeset if the manuscript is accepted for publication.<br><br>*PLOS allows rare exemptions to address legal and ethical concerns. If you have legal or ethical restrictions, please use the box below to detail these in full sentences for the Journal team to consider.*	

Powered by Editorial Manager® and ProduXion Manager® from Aries Systems Corporation

Manuscript

Click here to access/download;Manuscript;Intersectional Analysis PLOS Manuscript.docx

Intersectional Disparities in Mental Healthcare Utilization by Sex and Race/Ethnicity among US Adults: An NHANES Study

Author(s)

Lotenna Olisaeloka, MD, MSc[1,2]; Gentille Musengimana, MSc[1]; Esteban J. Valencia, MPH[1], Daniel Vigo, MD, Dr.PH[1,2]; Ehsan Karim, Ph.D[1]

1. School of Population and Public Health, University of British Columbia.
2. Department of Psychiatry, Faculty of Medicine, University of British Columbia.

Corresponding Author: Lotenna Olisaeloka

Contact:
School of Population and Public Health (SPPH),
2206 East Mall, Vancouver, British Columbia,
V6T 1Z3, Canada.

Keywords: Mental Health, Intersectionality, Race/Ethnicity, Sex, Health Disparities, Equity

ABSTRACT

Introduction: Mental healthcare utilization in the US remains low, with sociodemographic factors like sex and race/ethnicity influencing access across different population groups. However, how these factors interact to shape service utilization remains understudied.

Methods: Using data from the 2009–2018 cycles of the National Health and Nutrition Examination Survey (NHANES), we employed design-based log-binomial models to estimate the relative differences (prevalence ratios) of mental healthcare utilization across intersecting sex and race/ethnic groups. To assess the absolute differences, we used linear probability regression models to estimate the prevalence differences across these intersectional groups. We also conducted stratified analyses by education, income, health insurance, and depression status, to examine whether disparities persisted across socioeconomic and health-related subgroups.

Results: Overall, 9.1% of adults reported accessing mental health services in the past year. Hispanic males had the lowest utilization rates compared to Non-Hispanic (NH) White males, with an adjusted prevalence ratio (aPR) of 0.59 [95% CI: 0.47–0.73]. Among females, significant disparities were observed across all race/ethnic minority groups with NH Black and Hispanic females having significantly lower utilization rates compared to NH White females. Absolute prevalence differences mirrored the relative measures. Stratified analysis were largely consistent with primary results but revealed some variation across education, income and health insurance strata.

Discussion: These findings reflect how intersecting sociodemographic characteristics, specifically sex and race/ethnicity, influence utilization of mental health services. Stratified results suggest that socio-economic status may modify these disparities, pointing to the role played by systemic inequities. Hence, culturally informed strategies and wider structural interventions are needed to address these disparities. Future research should consider additional intersecting identities (e.g., sexual orientation and disability) and investigate socio-structural approaches to reducing these gaps.

INTRODUCTION

Mental health disorders affect approximately 1 in 8 people globally, with prevalence remaining high over the past three decades despite evidence-based treatment options[1–3]. This is partly attributable to a persistent mental health treatment gap, with over 75% of individuals experiencing mental health problems globally not receiving necessary care[4]. In the United States, the number of adults with any mental illness increased by 30% from 2008 to 2019, with over 50 million adults currently affected[5,6] Yet, more than half of them do not receive care[7,8]. Preventive mental health services like screening and early intervention are also critically lacking, especially for at-risk groups like individuals with chronic physical conditions[9]. Addressing these gaps and ensuring equitable access to mental healthcare presents a pressing public health challenge.

Social determinants of health such as gender, ethnicity, sexual orientation and socioeconomic status influence mental health and access to care[10–12]. Social inequities create barriers to obtaining quality mental healthcare and exacerbate health disparities. Race and ethnicity, in particular are recognized as social constructs shaped by historical, cultural and political factors rather than biological differences. These categories often reflect differential access to resources and opportunities, which influence healthcare access and outcomes[13]. While single-factor disparities by sex and race/ethnicity have been observed, with women and non-Hispanic Whites generally showing higher service utilization rates, the intersection of these factors remain understudied[11,14–18]. Intersectionality, a framework born out of black women's concurrent experiences of racism and sexism emphasizes the interconnected nature of social inequalities[13,19,20]. The rise of intersectionality in health equity research underscores the limitations of single-factor approaches which treat social categories as independent, and thus fail to capture how the complex interplay of factors shape disparities[19,21].

Although quantitative methods are crucial for identifying health disparities at the population level, intersectional research has remained largely qualitative due to challenges in translating theoretical constructs into statistical models[19]. This study addresses this gap in relation to mental healthcare utilization by operationalising intersectionality using regression models with interaction terms, as proposed by Bauer (2014)[19]. Specifically, we estimate disparities in mental healthcare utilization among U.S adults across intersections of sex and race/ethnicity. While two axes of identity are not

exhaustive, they represent critical starting points for operationalizing intersectionality in healthcare access research[19]. Identifying disproportionately affected intersectional groups can inform targeted interventions and health equity strategies.

METHODS

Data Source

We utilized aggregated data from five continuous cycles of the National Health and Nutrition Examination Survey (NHANES) from 2009-2018. The NHANES is an annual population-based, cross-sectional survey of civilian, non-institutionalized U.S. residents conducted by the Centers for Disease Control and Prevention (CDC)[22]. The survey collects sociodemographic, dietary, and health-related information from a nationally representative sample by employing a multi-stage probability sampling methodology. Detailed information on design, methodology and weighting are published on the CDC website. The NHANES receives ethical approval from the CDC Institutional Review Board and all participants gave informed consent[22]. This NHANES dataset is anonymous and publicly available, hence the study is exempt from institutional ethic review as outlined in the institution's and Tri-Council Policy Statements (TCPS2) on Ethical Conduct for Research Involving Humans[23,24]. We assessed the survey data from 3rd November, 2024 and did not have access to any information that could identify participants at any stage.

Study Sample

Aggregated data from the five NHANES cycles included 49,693 participants, of which only adults aged 18 years and over were included in the study (n = 30,352). Following the exclusion of individuals with missing values in exposure and outcome, the final analytical sample was 30,340. **Figure 1** illustrates the process of creating the analytical sample.

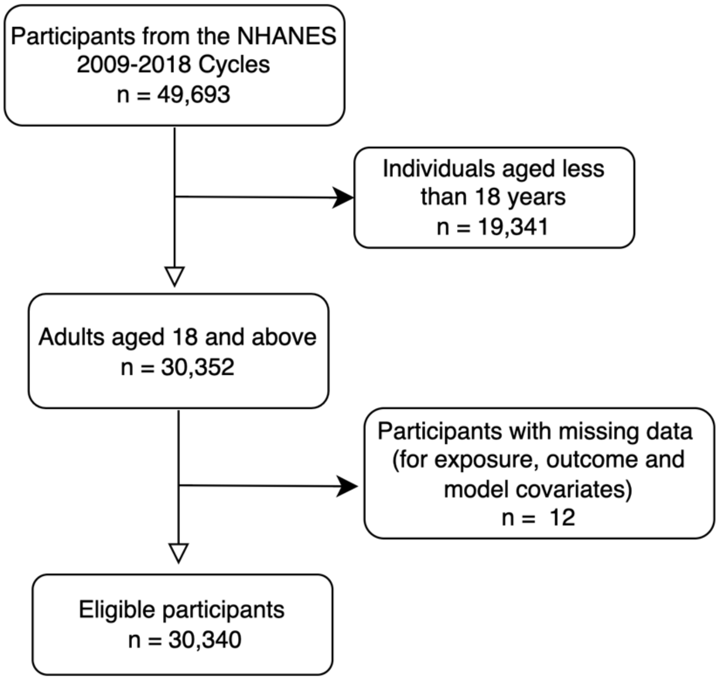

Figure 1: Flowchart illustrating the sample selection process of adults aged 18+ from the NHANES cycles.

Outcome

The outcome, 'mental healthcare utilization' reflected whether respondents accessed mental health services in the past twelve months and was ascertained by an affirmative response to the question: "*During the last 12 months, have you seen or talked to a mental health professional about your health?"* [25].

Exposure

The exposure variables were sex and race/ethnicity as collected by NHANES. Sex was categorized as male or female, and participants were grouped by race/ethnicity variable (RIDRETH1) into four categories (Non-Hispanic White, Hispanic, Non-Hispanic Black and Non-Hispanic Other Race). The 'Non-Hispanic Other Race' category included individuals who identified as Non-Hispanic Asian, American Indian or Alaska Native, Native Hawaiian or Other Pacific Islander, and multiracial individuals. This grouping reflects NHANES' racial and ethnic classifications and was used due to sample size limitations for more granular subgroup analysis.

While race, ethnicity, and sex are immutable characteristics, they serve as proxies for social experiences shaped by systemic factors such as structural racism, discrimination, and privilege. These variables were included to assess disparities in mental healthcare utilization, reflecting the influence of systemic and structural factors.

Covariate selection

We used a directed acyclic graph (DAG) using *Dagitty* (version 3.1)[26] to identify suitable risk factors for the outcome which were not positioned along the exposure-outcome causal pathway[27]. Based upon the literature, we considered the following factors: age, education level, marital status, income level, health insurance, perceived health status, depression and chronic physical condition [**Figure 2**][11,12,15,28–30]. Income level (low, middle, high) was categorized using recommended thresholds of poverty income ratio (PIR)[31]. Perceived health status refers to how participants rated their current general health status (NHANES variable HSD010)[22]. Depression was categorized by severity (none, mild, moderate, moderately severe and severe) based on the Patient Health Questionnaire-9 (PHQ-9) scores[32]. Chronic condition indicated whether participants had diabetes, hypertension, cancer or chronic kidney disease. Age emerged as the sole variable that did not introduce bias when adjusted for [**Figure 2**]. Accordingly, all adjusted models control for age as a precision variable.

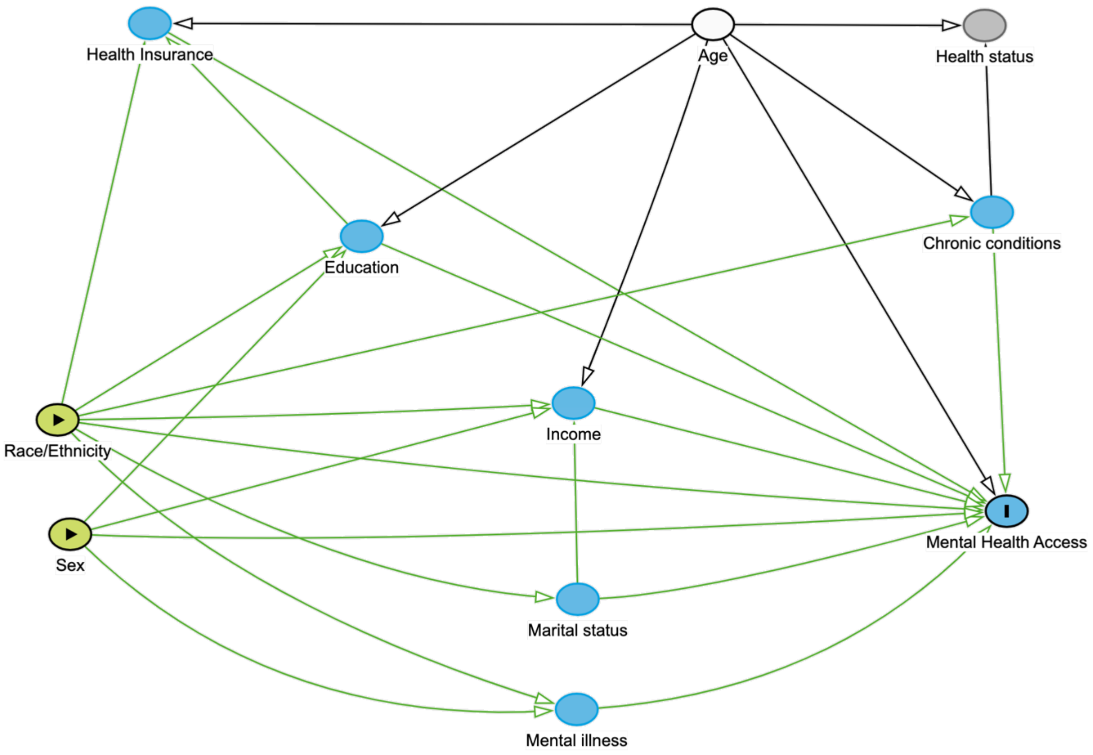

Figure 2: Directed acyclic graph of the relationships between sex, race/ethnicity and mental health service utilization.

Note: Sex and Race/ethnicity are represented as exposures. Mental health access is represented as the outcome. Solid, blue-shaded cells indicate possible mediators of the exposure-outcome relationship. Solid white shaded cells indicate independent risk factors for the outcome. The grey shaded cell indicates a collider variable.

Statistical Analysis

Descriptive statistics were conducted to summarize the study sample, with sociodemographic and health-related characteristics stratified by sex and race/ethnicity. Categorical variables were presented as counts with their respective weighted percentages. All analyses incorporated survey weights, clusters, and strata to account for the complex survey design[33].

Primary Analysis

To operationalize intersectionality in this quantitative analysis, we employed interaction terms between sex and race/ethnicity in our regression models. To examine whether utilization rates differed across intersectional groups, we used design-based log-binomial and linear probability models to estimate interaction on the multiplicative and additive scales respectively[19,34]. Prevalence ratios (PR) and Prevalence Differences (PD), with their 95% confidence intervals (95% CI) and significance levels, were obtained via simple slope/effects analysis. Overall missingness and missingness within variables in the model were less than 5% respectively, hence a complete case analysis was conducted[35].

Stratified/Sensitivity Analyses

To assess the consistency of the primary results as well as examine the role of socioeconomic and health-related factors in shaping the observed associations, we conducted stratified analyses by key variables: education level (high school and below vs. college and above), income level (low vs. middle vs. high), and health insurance status (insured vs. uninsured). Stratified analysis was also done by mental illness (depression – yes/no) to assess whether the observed results differed by mental healthcare need. Within each stratum, separate design-based log-binomial regression models including the interaction term between sex and race/ethnicity were fitted. This approach allowed us to extend the intersectional analysis beyond race/ethnicity and sex, evaluating whether the associations and interaction effects observed in the primary analysis differed across socioeconomic groups.

To address missingness in key stratification variables, such as income level (10.5%) and depression (14.2%), we conducted multiple imputation. Little's MCAR test ($p < 0.001$) and bivariate analyses respectively revealed that missingness was not completely at random (MCAR) but could be explained by observed data. Under the missing at random (MAR) assumption, our imputation model included variables from the primary model, those requiring imputation (income level, education level, health insurance, and depression), and auxiliary variables (annual family income, marital status, chronic condition, health status, and survey strata). Five imputations with 20 iterations each were performed using logistic regression for binary variables and polynomial regression for categorical variables. The number of iterations was determined by monitoring the trace plots until they demonstrated convergence. Individuals with missing outcome data (n=12)

were excluded, resulting in a final analytical sample of 30,340. Design-adjusted multivariable regression models were run on each imputed dataset, and results were pooled using Rubin's rule which combines the within- and between- imputation variances[36].

All statistical analyses were conducted using R (v4.3.1). The *survey* package was used to fit the design-adjusted models[37], and *mice* package was used for missing data analysis. This study followed the Strengthening the Reporting of Observational Studies in Epidemiology (STROBE) reporting guidelines as well as widely adopted recommendations for reporting on sex/gender and race/ethncity[38–40].

RESULTS

Sample Characteristics

The study sample consisted of 30,340 participants, with a nearly even distribution of males (48.5%) and females (51.5%). **Tables 1a and b** present the characteristics of the participants stratified by sex and race/ethnicity respectively. The counts were from the eligible sample while the percentages were derived using survey features, hence represent the national distribution. Significant sex and race-ethnic differences were observed across most demographic and health-related characteristics including education, income level, marital status and health insurance. The sample was predominantly Non-Hispanic (NH) White (64.8%), with small but significant differences in racial/ethnic composition between sexes. Overall, 9.1% of the U.S population accessed mental health services. Females reported a significantly higher proportion of mental health service utilization than males (9.7% vs 8.4%). Stratification by race/ethnicity revealed distinct differences in sociodemographic characteristics and health outcomes. For example, Hispanics had the highest proportion of low-income individuals (30.9%), and NH Whites were significantly more likely to report *excellent* and *very good* health compared to other race/ethnic groups. Mental healthcare utilization was highest among NH Whites (9.8%) and lowest among Hispanic and Other Race groups (7.3% each) [**Table 1b**].

Table 1a: Sample Characteristics by Sex among US adults aged 18 years or more in the 2009-2018 NHANES

Characteristic (%)	Overall 30,340	Male 14,707(48.5)	Female 15,633 (51.5)	p value
**Age**				
18-39	11,147 (38.4)	5,418 (39.7)	5,729 (37.2)	0.017
40-59	9,442 (35.5)	4,488 (35.9)	4,954 (35.2)	
60-79	7,810 (21.6)	3,887 (20.8)	3,923 (22.4)	
80+	1,941 (4.4)	914 (3.5)	1,027 (5.3)	
**Ethnicity**				
Non-Hispanic White	11,699 (64.8)	5,793 (65.2)	5,906 (64.5)	0.003
Hispanic	7,671 (15.0)	3,598 (15.6)	4,073 (14.5)	
Non-Hispanic Black	6,611 (11.5)	3,177 (10.7)	3,434 (12.3)	
Other race*	4,359 (8.6)	2,139 (8.6)	2,220 (8.7)	
**Marital status**				
Married/with partner	16,864 (62.5)	8,881 (65.8)	7,983 (59.5)	<0.001
Divorced/Separated	4,141 (12.7)	1,697 (10.6)	2,444 (14.7)	
Never married	5,453 (18.8)	2,769 (21.1)	2,684 (16.8)	
Widowed	2,345 (5.9)	590 (2.5)	1,755 (9.0)	
**Education Level**				
High School or Less	7,235 (15.6)	3,672 (16.4)	3,563 (14.9)	<0.001
High School Graduate	7,447 (24.1)	3,780 (25.4)	3,667 (22.9)	
Some College	8,639 (30.8)	3,843 (28.7)	4,796 (32.7)	
College Graduate or Above	6,857 (29.5)	3,334 (29.4)	3,523 (29.6)	
**Income**				
Low Income	6,413 (15.8)	2,843 (14.2)	3,570 (17.03)	<0.001
Middle Income	14,157 (48.6)	6,937 (47.9)	7,220 (49.1)	
High Income	65,888 (35.6)	3,400 (37.8)	3,188 (33.6)	
**Mental Healthcare Utilization**				
No	27,753 (90.9)	13,567 (91.6)	14,186 (90.3)	<0.001
Yes	2,587 (9.1)	1,140 (8.4)	1,447 (9.7)	

**Depression**				
None	1,9471 (76.4)	10,177 (80.8)	9,294 (72.3)	<0.001
Mild	4,220 (15.5)	1,772 (13.3)	2,448 (17.7)	
Moderate	1,443 (5.1)	544 (3.7)	899 (6.4)	
Severe	904 (3.0)	316 (2.2)	588 (3.7)	
**Chronic Conditions**				
No	15,662 (58.5)	7,607 (59.0)	8,055 (58.1)	0.485
Yes	13,194 (41.5)	6,353 (41.0)	6,841 (41.9)	
**Health status**				
Excellent	1,879 (8.7)	1,031 (9.0)	848 (8.3)	<0.001
Very good	6,830 (32.5)	3,393 (31.3)	3,437 (33.7)	
Good	10,773 (40.5)	5,326 (41.8)	5,447 (39.2)	
Fair	5,407 (15.7)	2,569 (15.7)	2,838 (15.8)	
Poor	966 (2.6)	396 (2.1)	570 (3.0)	
**Health insurance**				
No	6,278 (17.2)	3,372 (19.5)	2,906 (15.0)	<0.001
Yes	24,004 (82.8)	11,310 (80.5)	12,694 (85.0)	

***** *Other race* includes Non-Hispanic Asians, individuals of other racial/ethnic identity (e.g., American Indian) and multiracial individuals.

Table 1b - Sample Characteristics stratified by Race-ethnicity among US adults aged 18 years or more in the 2009-2018 NHANES

Level (%)	Overall	Non-Hispanic White	Hispanic	Non-Hispanic Black	Other race	p-value
N	3,0340	11,699(64.8)	7,671(15.0)	6,611(11.5)	4,359 (8.6)	
**Sex**						0.003
Male	14,707 (48.2)	5,793 (48.5)	3,598 (50.0)	3,177(44.7)	2,139 (47.8)	
Female	15,633 (51.8)	5,906 (51.5)	4,073 (50.0)	3,434 (55.3)	2,220 (52.2)	
**Age**						<0.001
18-39	11,147 (38.4)	3,886 (33.4)	3,040 (52.6)	2,367 (43.3)	1,854 (44.6)	

40-59	9,442 (35.5)	3,382 (35.9)	2,433 (33.4)	2,132 (36.1)	1,495 (35.4)	
60-79	7,810 (21.6)	3,065 (25.0)	2,004 (12.5)	1,867 (18.0)	874 (17.4)	
80+	1,941 (4.4)	1,366 (5.7)	194 (1.5)	245 (2.6)	136 (2.6)	
**Marital status**						<0.001
Divorced/Separated	4,141 (12.7)	1,607 (12.5)	1,062 (12.8)	1,141 (16.6)	331 (9.0)	
Married/with partner	16,864 (62.5)	6,842 (65.2)	4,550 (64.2)	2,686 (42.6)	2,786 (66.0)	
Never married	5,453 (18.8)	1,693 (15.7)	1,096 (19.4)	1,865 (34.2)	799 (20.9)	
Widowed	2,345 (5.9)	1,139 (6.5)	460 (3.6)	551 (6.6)	195 (4.1)	
**Education level**						<0.001
High School or Less	7,235 (15.6)	1,795 (10.1)	3,384 (38.4)	1,373 (18.8)	683 (13.9)	
High School Graduate	7,447 (24.1)	2,999 (23.9)	1,745 (24.8)	1,898 (29.1)	805 (18.1)	
Some College	8,639 (30.8)	3,754 (32.0)	1,689 (24.7)	2,195 (34.3)	1,001 (27.1)	
College Graduate or above	6,857 (29.5)	3,121 (34.1)	788 (12.1)	1,093 (17.8)	1,855 (40.9)	
**Income Level**						<0.001
Low Income	6,413 (15.8)	1,902 (10.4)	2,142 (30.9)	1,647 (27.9)	722 (16.6)	
Middle Income	14,157 (48.6)	5,692 (46.4)	3,546 (54.2)	3,072 (52.7)	1,847 (50.2)	
High Income	6,588 (35.6)	3,329 (43.1)	874 (14.8)	1,112 (19.5)	1,273 (33.2)	
**Mental Healthcare Utilization**						<0.001
No	27,753 (90.9)	10,522(90.2)	7,123 (92.7)	6,016 (91.1)	4,092 (92.7)	
Yes	2,587 (9.1)	1,177 (9.8)	548 (7.3)	595 (8.9)	267 (7.3)	
**Chronic conditions**						<0.001
No	15,662 (58.5)	5,790 (56.2)	4,268 (68.7)	2,989 (54.1)	2,615 (64.5)	
Yes	13,194 (41.5)	5,499 (43.8)	2,903 (31.3)	3,286 (45.9)	1,506 (35.5)	
**Depression**						<0.001
None	19,471 (76.4)	7,672 (77.1)	4,806 (74.9)	4,218 (73.7)	2,775 (77.7)	
Mild	4,220 (15.5)	1,700 (15.3)	1,906 (16.1)	942 (17.0)	482 (14.3)	
Moderate	1,443 (5.1)	594 (4.9)	389 (5.3)	315 (5.9)	145 (5.5)	
Severe	904 (3.0)	368 (2.8)	278 (3.6)	189 (3.4)	69 (2.6)	
**Health status**						<0.001

Excellent	2,416 (10.5)	1,059 (11.4)	489 (8.1)	462 (8.4)	406 (10.9)	
Very good	6,830 (31.9)	3,447 (36.5)	1,065 (18.7)	1,303 (23.5)	1,015 (29.4)	
Good	10,773 (39.7)	4,063 (38.2)	2,743 (42.7)	2,446 (42.5)	1,521 (42.2)	
Fair	5,407 (15.4)	1,562 (11.9)	2,016 (26.5)	1,326 (22.3)	503 (14.8)	
Poor	966 (2.5)	335 (2.0)	341 (4.1)	208 (3.3)	82 (2.7)	
**Health insurance**						<0.001
No	6,278 (17.2)	1,638 (11.5)	2,666 (38.4)	1,265 (21.8)	709 (16.8)	
Yes	24,004 (82.8)	10,049(88.5)	4,986 (61.6)	5,329 (78.2)	3,640 (83.2)	

Mental Healthcare Utilization across Intersectional Groups

We observed significant disparities in relative and absolute prevalence estimates of mental healthcare utilization across sex and race/ethnic intersectional groups **[Table 2].** Compared to NH White males, Hispanic males were the least likely (41% less likely, aPR = 0.59 [0.47–0.73]) to utilize services, followed by males in the Other race group (33% less likely, aPR = 0.66 [0.50–0.88]). NH Black males were also less likely to utilize mental health services compared to NH White males. However, this association was insignificant (aPR = 0.91 [0.75–1.12]). Among females, Hispanic, NH Blacks and women in the Other race category were significantly less likely (25%, 19% and 27% less likely respectively) to utilize mental health services compared to NH White females.

Within the same race-ethnic group, females were more likely to utilize services than males. While NH White (aPR = 1.16 [1.00, 1.36]) and Hispanic females (aPR = 1.49 [1.22, 1.80]) had significantly higher probabilities of utilizing services than their male counterparts, this association was not significant among NH Blacks and individuals in the Other race category.

Results on the absolute/additive scale were consistent with the relative findings but indicated smaller differences [**Table 2**].

Table 2: Prevalence Ratios (PR) and Prevalence Differences (PD) of Mental Healthcare Utilization by Sex and Race/Ethnicity (95% Confidence Intervals)

**Race/Ethnicity**<br>**PR & PD (95% CI)**	**Male**	**Female**	**Female vs Male** (within strata of race-ethnicity)
**Relative Scale**			

**Unadjusted PR**			
Non-Hispanic White	Ref^	Ref	1.14 (0.97, 1.32)
Hispanic	0.65 (0.52, 0.80)***	0.84 (0.70, 0.99)*	1.46 (1.20, 1.79)***
Non-Hispanic Black	0.97 (0.80, 1.19)	0.87 (0.73, 1.05)	1.02 (0.87, 1.20)
Other Race	0.70 (0.53, 0.92)**	0.79 (0.61, 1.00)*	1.27 (0.92, 1.75)
**Adjusted PR**			
Non-Hispanic White	Ref	Ref	1.16 (1.00, 1.36)*
Hispanic	0.59 (0.47, 0.73)***	0.75 (0.63, 0.90)***	1.49 (1.22, 1.80)**
Non-Hispanic Black	0.91 (0.75, 1.12)	0.81 (0.68, 0.98)*	1.04 (0.89, 1.21)
Other Race	0.66 (0.50, 0.88)**	0.73 (0.57, 0.92)*	1.27 (0.92, 1.77)
**Absolute Scale**			
**Unadjusted PD**			
Non-Hispanic White	Ref	Ref	0.01 (-0.00, 0.03)
Hispanic	-0.03 (-0.05, -0.02)***	-0.02 (-0.03, -0.00)*	0.03 (0.01, 0.04)***
Non-Hispanic Black	-0.00 (-0.02, 0.02)	-0.01 (-0.03, 0.00)	0.00 (-0.01, 0.02)
Other Race	-0.03 (-0.05, -0.01)**	-0.02 (-0.04, -0.00)*	0.02 (-0.01, 0.04)
**Adjusted PD**			
Non-Hispanic White	Ref	Ref	0.01 (-0.00, 0.03)
Hispanic	-0.03 (-0.05, -0.01)***	-0.02 (-0.03, -0.00)**	0.03 (0.01, 0.04)***
Non-Hispanic Black	-0.00 (-0.02, 0.01)	-0.02 (-0.03, 0.00)	-0.00 (-0.01, 0.01)
Other Race	-0.03 (-0.05, -0.01)**	-0.02 (-0.04, -0.00)*	0.02 (-0.01, 0.04)

Adjusted estimates are adjusted for Age.
^Ref are the reference groups of comparisons. Within male and female categories, individuals in other intersectional groups are compared to Non-Hispanic Whites.
*indicates statistical significance at p values: *<0.05, **<0.01 ***<0.001

Stratified Analysis

The results of the stratified analyses were largely consistent with the primary analysis, although few subgroup differences emerged across education, income, health insurance, and depression status. [**Table 3**].

Education

Compared to the primary analysis, stratification by education revealed notable differences among NH Black individuals. Among those with *high school education or less*, NH Black males had higher mental health service utilization than NH White males (aPR = 1.26, 95% CI: 0.96–1.65), contrasting with the overall finding of lower utilization among NH Black males in the primary

analysis. NH Black males with *high school education or less* were also more likely to utilize services compared to their female counterparts (NH Black females) [**Table 3**]. Although these differences were not statistically significant, they indicate a directional reversal in disparities among lower-educated NH Black individuals compared to results from the primary analysis. Conversely, among those with higher education (*college and above*), findings mirror the main analysis with NH Black males having significantly lower utilization than NH White males, and NH Black females having higher utilization than NH Black males.

Income

Among individuals in the *middle-income* category, results from the stratified analysis aligned closely with the overall findings. However, among those with *low income*, NH Black females had lower utilization than NH Black males (aPR = 0.72 [0.52, 0.99]), again reversing the sex pattern from the primary analysis. This reversal is consistent with the pattern seen in the lower education stratum, suggesting a potential intersection of sex and socio-economic status in shaping service use. Among individuals with high-income, disparities across intersectional groups were not statistically significant. This contrasts with the significant disparities seen in the main analysis, suggesting that higher income may attenuate disparities.

Insurance

Stratified results by insurance status were broadly consistent with the primary analysis, particularly in terms of direction. However, disparities were more pronounced among uninsured females, with ethnic minority females having even lower utilization rates than in the main analysis. For example, uninsured Hispanic women had 70% lower utilization than uninsured NH White women (aPR = 0.30, 95% CI: 0.21–0.41), a sharper disparity than observed in the primary analysis (25% lower utilization, aPR = 0.75), indicating that lack of insurance may exacerbate disparities among women.

Depression

The adjusted prevalence ratios (aPRs) for mental healthcare utilization stratified by depression status were largely consistent with the primary analysis suggesting that depression status did not substantially alter the observed disparities.

Table 3: **Results of Stratified Analysis by Education level, Income level, Health Insurance and Depression status showing adjusted Prevalence Ratios (PR) with 95% CIs.**

Stratification Variable	Male<br>aPR (95% CI)	Female<br>aPR (95% CI)	Female vs Male<br>aPR (95% CI)
**Education**			
**High School or below**			
Non-Hispanic White	Ref	Ref	1.17 [0.91; 1.51]
Hispanic	0.58 [0.44,0.75]***	0.74 [0.58,0.93]**	1.51 [1.17,1.92]***
Non-Hispanic Black	1.26 [0.96, 1.65]	0.93 [0.72, 1.21]	0.87 [0.68; 1.12]
Other race	0.89 [0.59, 1.35]	0.94 [0.64, 1.38]	1.25 [0.73, 2.12]
**College and above**			
Non-Hispanic White	Ref	Ref	1.14[0.96, 1.35]
Hispanic	0.73 [0.54,0.99]*	0.88[0.69,1.12]	1.38[1.02; 1.88]*
Non-Hispanic Black	0.70 [0.55, 0.88]**	0.78[0.63, 0.97]*	1.28[0.99; 1.67]
Other race	0.58[0.39, 0.86]**	0.66 [0.50, 0.87]**	1.31[0.83, 2.06]
**Income**			
**Low Income**			
Non-Hispanic White	Ref	Ref	1.22 [0.99, 1.51]
Hispanic	0.46 [0.36, 0.60]***	0.47[0.36, 0.62]***	1.01 [0.73, 1.39]
Non-Hispanic Black	1.01 [0.77, 1.31]	0.67 [0.50, 0.99]**	0.72 [0.52, 0.99]
Other race	0.68 [0.43, 1.10]	0.62 [0.44, 0.90]**	1.06 [0.64, 1.75]
**Middle Income**			
Non-Hispanic White	Ref	Ref	1.15 [0.89, 1.49]
Hispanic	0.56 [0.40, 0.79]***	0.75[0.46, 1.06]	1.62 [1.20, 2.18]**
Non-Hispanic Black	0.75 [0.55, 1.03]	0.75 [0.52, 1.57]	1.26 [0.93, 1.72]
Other race	0.45[0.28, 0.75]***	0.57 [0.29, 1.14]	1.65 [0.95, 2.82]
**High Income**			
Non-Hispanic White	Ref	Ref	1.11 [0.84, 0.46]
Hispanic	0.73 [0.46, 1.15]	1.00[0.63, 0.90]	1.54 [0.89, 2.67]
Non-Hispanic Black	0.72 [0.49, 1.06]	0.95 [0.57, 1.57]	0.95 [0.57, 1.57]
Other race	0.76 [0.43, 1.36]	0.89 [0.44, 1.73]	0.87 [0.44, 1.73]
**Insurance**			
**Uninsured**			

Non-Hispanic White	Ref	Ref	1.75[1.21; 2.53]**
Hispanic	0.52 [0.34,0.81]**	0.30 [0.21,0.41]***	1.00 [0.73,1.37]
Non-Hispanic Black	0.87[0.54, 1.37]	0.45[0.30,0.70]***	0.92[0.59; 1.43]
Other race	0.73 [0.33, 1.62]	0.43[0.25, 0.76]**	1.04[0.47, 2.32]
**Insured**			
Non-Hispanic White	Ref	Ref	1.11[0.94, 1.28]
Hispanic	0.73 [0.57,0.94]*	1.00[0.84,1.20]	1.51[1.20; 1.92]***
Non-Hispanic Black	0.99[0.80, 1.12]	0.90[0.74, 1.08]	1.00[0.86; 1.17]
Other race	0.67[0.49, 0.90]**	0.78[0.79, 1.01]	1.28[0.89, 1.84]
**Depression status**			
**No Depression**			
Non-Hispanic White	Ref	Ref	1.08[0.86; 1.37]
Hispanic	0.55[0.42,0.72]***	0.75[0.57,0.98]*	1.48[1.13,1.91]**
Non-Hispanic Black	0.88[0.66, 1.17]	0.73[0.52, 0.90]**	0.84[0.66; 1.09]
Other race	0.57 [0.34, 0.93]*	0.73 [0.52, 1.04]	1.40[0.80, 2.46]
**Depression**			
Non-Hispanic White	Ref	Ref	0.99[0.81, 1.21]
Hispanic	0.61[0.46,0.81]***	0.70[0.57,0.87]***	1.14[0.89; 1.46]
Non-Hispanic Black	0.90[0.70, 1.15]	0.84[0.68, 1.03]	0.92[0.76; 1.12]
Other race	0.82[0.57, 1.17]	0.76 [0.57, 1.01]	0.91[0.62, 1.37]

Discussion

Main Findings

This study examined disparities in mental healthcare utilization among U.S adults across intersections of sex and race-ethnicity using data from NHANES 2009-2018 cycles. Overall, only 9.1% of adults in the United States reported accessing mental health services in the past year, which aligns with existing research documenting low utilization rates despite high prevalence of mental health needs[7,8]. The primary interaction analysis demonstrated that mental healthcare utilization varied significantly across intersecting sex and race/ethnicity groups. Among males, Hispanic men were the least likely (41% less likely) to access mental healthcare relative to NH White men. Similarly, females across all minority race/ethnicity groups were significantly less

likely to access services than NH White females, with prevalence ratios ranging from 0.75 to 0.81. Within the same racial/ethnic groups, females generally accessed services more than their male counterparts. Prevalence estimates on the absolute scale aligned with findings on the relative scale although the absolute differences were smaller which possibly reflects the low overall prevalence of mental healthcare utilization in the sample.

Study findings generally align with existing literature that show females having high utilization rates than males and NH whites having higher rates than other race/ethnic groups. However, by applying an intersectional framework and modelling prevalence estimates on relative and absolute scales, this analysis captured nuanced disparities that might otherwise remain obscured by single factor approaches[11,17,21]. For instance, Hispanic males demonstrated the lowest utilization rates, representing a crucial disparity point requiring targeted intervention[41]. Further, the varying magnitude of sex gaps across race-ethnic groups—from 49% higher rates for Hispanic females to only 4% for NH Black females compared to their male counterparts—suggests that sex and race-ethnicity interact differently across groups.

The observed disparity patterns likely reflect complex interactions between social norms, help-seeking behaviors, and systemic factors rather than differences in mental health diagnosis or needs. Research shows that minority ethnic groups like Hispanics and NH Blacks have similar or higher prevalence of mental disorders compared to NH Whites[18,42,43]. This is also reflected in this study sample in which Hispanics and NH Blacks have higher prevalence of depression severity than NH Whites. Further, the prevalence ratios for mental healthcare utilization across depression strata were largely consistent with the main analysis, suggesting that disparities in access persist regardless of mental health needs. This finding aligns with Crenshaw's intersectionality theory and existing research on how intersecting social identities, rather than clinical need alone, influence healthcare access and utilization[18,20,30].

Several factors might contribute to these observed intersectional disparities. Cultural and systemic barriers play significant roles, including limited cultural competence among healthcare providers, varying cultural conceptualizations of mental health and illness, and differential stigma across cultural groups[44,45]. Additionally, structural barriers such as economic disparities, and insurance coverage gaps likely contribute to these patterns[5]. For instance, the pronounced disparities among

Hispanic males might reflect the intersection of traditional masculine norms alongside barriers disproportionately affecting Hispanics including language barriers and immigration-related issues such as being undocumented, working low-income jobs, and not having health insurance[41,44,46–50].

Stratified Analysis

In the stratified analysis, some differences observed among some subgroups highlight how socioeconomic factors might modulate mental health access disparities. For example, lack of health insurance amplified disparities across sex and race/ethnicity, particularly for Hispanic and NH Black females. Likewise, income level seemingly influenced mental healthcare utilization, particularly for NH Blacks and Hispanics. Additionally, the absence of significant differences among high-income individuals suggests that socioeconomic advantages may reduce disparities observed in the overall sample.

Education also appeared to influence mental healthcare utilization rates particularly among NH Blacks. In contrast to the primary results and findings from individuals in the higher education category, NH Black males with low education had higher utilization rates than NH White males. Additionally, NH Black females in this group were significantly less likely to access services than NH Black males. This suggests that the overall non-significant difference in utilization observed for NH Black males compared to NH White males and NH Black females may be due to the higher service utilization among NH Black males with lower education. This finding possibly reflects diagnostic patterns, as Black men, particularly those with lower education and socioeconomic status are more likely than White men and Black women to be diagnosed with severe mental illness and substance use disorder[42,45]. Consequently, they are more likely to enter treatment through involuntary or mandated pathways including criminal justice referrals and emergency services rather than voluntary help-seeking[42,45,51].

Policy and Practice Implications

This study's findings have important implications for public health practice and policy. They suggest the need to better understand reasons for the observed disparities and for targeted multi-level interventions that address both socio-cultural and structural barriers to mental health service utilization. At the healthcare system level, increasing diversity and cultural competency of mental healthcare professionals, and implementing culturally informed mental health interventions could

address the unique needs and preferences of different intersectional groups, and help reduce disparities. Community-level interventions might include engaging community leaders in mental health outreach, developing culturally specific anti-stigma campaigns, and creating targeted programs for underserved intersectional groups, particularly Hispanic males[28,42,50,52]. Wider structural reforms are also essential to address socioeconomic barriers to mental healthcare. Expanding Medicaid and other insurance programs can reduce cost-related access barriers, while mobile clinics and telehealth programs, can help reach individuals in underserved areas[5,52,53]. Policies addressing social determinants of health including education, employment, and housing are also critical for achieving equitable access to care[52,54]. Addressing these intersectional disparities is critical not only for improving population mental health outcomes but also for broader public health and socioeconomic benefits, including enhanced productivity and increased quality of life[55].

Limitations

Several limitations should be considered when interpreting these findings. First, NHANES' use of binary sex assigned at birth rather than gender identity represents a significant limitation[25]. While sex and gender are correlated, they are distinct constructs. Thus, this limitation masks important nuances in mental health service utilization among gender-diverse populations. This is particularly important as research shows that transgender and non-binary individuals experience disproportionately higher rates of mental health challenges and face unique barriers to care[56].

Second, intersectionality is not limited to sex or gender and race/ethnicity but also include other characteristics like sexual orientation which could not be included in this analysis despite being important given well-documented mental health disparities among LGBTQ+ populations[21,56]. This limitation stemmed from methodological challenges in the NHANES dataset including insufficient sample sizes for non-heterosexual groups even after combining multiple cycles and high rates of non-specific responses to sexual orientation questions (e.g., ‘don’t know’, ‘refused’, ‘not sure’ or ‘something else’). Thus, the National Centre for Health Statistics discourages the use of this variable for analysis[22]. This reflects a broader challenge in population health data systems, highlighting the need for improved data collection methods that better capture sexual orientation while maintaining statistical power for meaningful analysis. Future research should prioritize the

inclusion of more comprehensive sociodemographic variables to capture the complexity of intersecting identities. Surveys and data collection tools could be enhanced by integrating better measures for gender identity, sexual orientation and disability.

Additional limitations include reliance on self-reported service utilization, which may be subject to recall and social desirability bias. Further, the exclusion of institutionalized populations from NHANES may miss important segments of the population needing mental health care. Despite these inherent limitations, this study leveraged an intersectional approach and utilized a large nationally representative sample to explore disparities in mental healthcare utilization across intersections of sex and race/ethnicity with additional consideration of other axes via stratified analysis.

Conclusion

Our findings reveal significant intersectional disparities in mental health service utilization in the United States, particularly by sex and race/ethnicity. These disparities are further shaped by socioeconomic factors such as education and income. These results underscore the need to consider multiple, intersecting identities when designing policies and interventions to improve mental healthcare access. Addressing these layered inequities will require both culturally responsive approaches and structural reforms to reduce barriers and promote equitable access to care.

Authors Contribution(s): L.O. conceptualized the study, developed the methodology, conducted the formal analysis, curated the data, wrote the original draft and revised the manuscript. G.M. contributed to the methodology, analysis and drafting of the manuscript. E.V. and E.K. assisted with analysis, writing, editing, and revision. D.V. contributed to revision and supervision. All authors reviewed and approved the final manuscript.

Competing Interests: No funding was sought for this research. Authors declare not conflict of interest.

References

1. Dattani S, Rodés-Guirao L, Ritchie H, Roser M. Mental Health. Our World Data [Internet]. 2023 Dec 28 [cited 2024 Jun 24]; Available from: https://ourworldindata.org/mental-health

2. Global, regional, and national burden of 12 mental disorders in 204 countries and territories, 1990–2019: a systematic analysis for the Global Burden of Disease Study 2019. Lancet Psychiatry. 2022 Feb 1;9(2):137–50.

3. Kessler RC, Demler O, Frank RG, Olfson M, Pincus HA, Walters EE, et al. Prevalence and Treatment of Mental Disorders, 1990 to 2003. N Engl J Med [Internet]. 2005 Jun 16 [cited 2024 Nov 7];352(24):2515–23. Available from: https://www.nejm.org/doi/full/10.1056/NEJMsa043266

4. Patel V, Maj M, Flisher AJ, Silva MJD, Koschorke M, Prince M, et al. Reducing the treatment gap for mental disorders: a WPA survey. World Psychiatry. 2010 Oct;9(3):169.

5. Exploring Barriers to Mental Health Care in the U.S. [Internet]. Research and Action Institute. [cited 2024 Dec 8]. Available from: https://www.aamcresearchinstitute.org/our-work/issue-brief/exploring-barriers-mental-health-care-us

6. 2023 National Survey on Drug Use and Health (NSDUH) Releases [Internet]. [cited 2024 Dec 8]. Available from: https://www.samhsa.gov/data/release/2023-national-survey-drug-use-and-health-nsduh-releases

7. Mental Illness - National Institute of Mental Health (NIMH) [Internet]. [cited 2024 Nov 13]. Available from: https://www.nimh.nih.gov/health/statistics/mental-illness

8. Kohn R, Ali AA, Puac-Polanco V, Figueroa C, López-Soto V, Morgan K, et al. Mental health in the Americas: an overview of the treatment gap. Rev Panam Salud Pública [Internet]. 2018 [cited 2024 Nov 13];42. Available from: http://iris.paho.org/xmlui/handle/123456789/49540

9. Liao B, Xu D, Tan Y, Chen X, Cai S. Association of mental distress with chronic diseases in 1.9 million individuals: A population-based cross-sectional study. J Psychosom Res. 2022 Nov 1;162:111040.

10. Compton MT, Shim RS. The Social Determinants of Mental Health. Focus. 2015 Oct;13(4):419–25.

11. Dobalian A, Rivers PA. Racial and Ethnic Disparities in the Use of Mental Health Services. J Behav Health Serv Res. 2008 Apr 1;35(2):128–41.

12. Wang J, Qiu Y, Zhu X. Trends of mental health care utilization among US adults from 1999 to 2018. BMC Psychiatry. 2023 Sep 12;23:665.

13. Funer F. Admitting the heterogeneity of social inequalities: intersectionality as a (self-)critical framework and tool within mental health care. Philos Ethics Humanit Med [Internet]. 2023 Nov 24 [cited 2024 Nov 7];18(1):21. Available from: https://doi.org/10.1186/s13010-023-00144-6

14. Cook BL, McGuire T, Miranda J. Measuring Trends in Mental Health Care Disparities, 2000–2004. Psychiatr Serv. 2007 Dec;58(12):1533–40.

15. Walker ER, Cummings JR, Hockenberry JM, Druss BG. Insurance Status, Use of Mental Health Services, and Unmet Need for Mental Health Care in the United States. Psychiatr Serv. 2015 Jun;66(6):578–84.

16. Byhoff E, Dinh DH, Lucas JA, Marino M, Heintzman J. Mental Health Care Use by Ethnicity and Preferred Language in a National Cohort of Community Health Center Patients. Psychiatr Serv Wash DC. 2024 Apr 1;75(4):363–8.

17. Hahm HC, Cook BL, Ault-Brutus A, Alegría M. Intersection of Race-Ethnicity and Gender in Depression Care: Screening, Access, and Minimally Adequate Treatment. Psychiatr Serv. 2015 Mar;66(3):258–64.

18. Manuel JI. Racial/Ethnic and Gender Disparities in Health Care Use and Access. Health Serv Res. 2018;53(3):1407–29.

19. Bauer GR. Incorporating intersectionality theory into population health research methodology: Challenges and the potential to advance health equity. Soc Sci Med [Internet]. 2014 Jun 1 [cited 2024 Nov 7];110:10–7. Available from: https://www.sciencedirect.com/science/article/pii/S0277953614001919

20. Crenshaw K. Demarginalizing the Intersection of Race and Sex: A Black Feminist Critique of Antidiscrimination Doctrine, Feminist Theory and Antiracist Politics. In: Feminist Legal Theories. Routledge; 1997.

21. Fagrell Trygg N, Månsdotter A, Gustafsson PE. Intersectional inequalities in mental health across multiple dimensions of inequality in the Swedish adult population. Soc Sci Med. 2021 Aug 1;283:114184.

22. NHANES Survey Methods and Analytic Guidelines [Internet]. [cited 2024 Nov 8]. Available from: https://wwwn.cdc.gov/Nchs/Nhanes/AnalyticGuidelines.aspx

23. Human Research Policy (LR9) | Office of the University Counsel [Internet]. [cited 2024 Dec 9]. Available from: https://universitycounsel.ubc.ca/policies/human-research-policy/

24. Government of Canada IAP on RE. Tri-Council Policy Statement: Ethical Conduct for Research Involving Humans – TCPS 2 (2022) [Internet]. 2023 [cited 2024 Nov 14]. Available from: https://ethics.gc.ca/eng/policy-politique_tcps2-eptc2_2022.html

25. NHANES Questionnaires, Datasets, and Related Documentation [Internet]. [cited 2024 Sep 14]. Available from: https://wwwn.cdc.gov/nchs/nhanes/

26. DAGitty v3.1 [Internet]. [cited 2024 Nov 15]. Available from: https://dagitty.net/dags.html#

27. VanderWeele TJ. Principles of confounder selection. Eur J Epidemiol. 2019 Mar 1;34(3):211–9.

28. Stewart-Brown S, Samaraweera PC, Taggart F, Kandala NB, Stranges S. Socioeconomic gradients and mental health: implications for public health. Br J Psychiatry [Internet]. 2015 Jun [cited 2024 Nov 13];206(6):461–5. Available from: https://www.cambridge.org/core/journals/the-british-journal-of-psychiatry/article/socioeconomic-gradients-and-mental-health-implications-for-public-health/F11FB267D70812FF5C014FD03EFDFAC7

29. Erving CL, Frazier C. THE ASSOCIATION BETWEEN MULTIPLE CHRONIC CONDITIONS AND DEPRESSIVE SYMPTOMS: DISTINCTIONS BY RACE, GENDER, AND AGE. Innov Aging [Internet]. 2019 Nov 8 [cited 2024 Nov 13];3(Supplement_1):S314. Available from: https://doi.org/10.1093/geroni/igz038.1148

30. Byers AL, Lai AX, Nelson C, Yaffe K. Predictors of Mental Health Services Use Across the Life Course among Racially–Ethnically Diverse Adults. Am J Geriatr Psychiatry. 2017 Nov 1;25(11):1213–22.

31. Jackson SL, Yang EC, Zhang Z. Income Disparities and Cardiovascular Risk Factors Among Adolescents. Pediatrics. 2018 Nov 1;142(5):e20181089.

32. Kroenke K, Spitzer RL, Williams JBW. The PHQ-9. J Gen Intern Med. 2001 Sep;16(9):606–13.

33. Fitting Regression Models to Survey Data on JSTOR [Internet]. [cited 2024 Nov 15]. Available from: https://www.jstor.org/stable/26408229

34. Naimi AI, Whitcomb BW. Estimating Risk Ratios and Risk Differences Using Regression. Am J Epidemiol. 2020 Jun 1;189(6):508–10.

35. Jakobsen JC, Gluud C, Wetterslev J, Winkel P. When and how should multiple imputation be used for handling missing data in randomised clinical trials – a practical guide with flowcharts. BMC Med Res Methodol. 2017 Dec 6;17(1):162.

36. Rubin DB. Multiple Imputation for Nonresponse in Surveys [Internet]. 1st ed. Wiley; 1987 [cited 2024 Dec 9]. (Wiley Series in Probability and Statistics). Available from: https://onlinelibrary.wiley.com/doi/book/10.1002/9780470316696

37. R: The R Project for Statistical Computing [Internet]. [cited 2024 Mar 22]. Available from: https://www.r-project.org/

38. Cuschieri S. The STROBE guidelines. Saudi J Anaesth. 2019 Apr;13(Suppl 1):S31–4.

39. Clayton JA, Tannenbaum C. Reporting Sex, Gender, or Both in Clinical Research? JAMA. 2016 Nov 8;316(18):1863–4.

40. Flanagin A, Frey T, Christiansen SL, AMA Manual of Style Committee. Updated Guidance on the Reporting of Race and Ethnicity in Medical and Science Journals. JAMA. 2021 Aug 17;326(7):621–7.

41. Chang CW, Biegel DE. Factors affecting mental health service utilization among Latino Americans with mental health issues. J Ment Health. 2018 Nov 2;27(6):552–9.

42. McGuire TG, Miranda J. Racial and Ethnic Disparities in Mental Health Care: Evidence and Policy Implications. Health Aff Proj Hope. 2008 Apr;27(2):393.

43. Breslau J, Kendler KS, Su M, Gaxiola-Aguilar S, Kessler RC. Lifetime risk and persistence of psychiatric disorders across ethnic groups in the United States. Psychol Med. 2005 Apr;35(3):317–27.

44. Villatoro A, Mays V, Ponce N, Aneshensel C. Perceived Need for Mental Health Care: The Intersection of Race, Ethnicity, Gender, and Socioeconomic Status. Soc Ment Health. 2018;8:1–24.

45. Hatzenbuehler ML, Keyes KM, Narrow WE, Grant BF, Hasin DS. Racial/Ethnic Disparities in Service Utilization for Individuals With Co-Occurring Mental Health and Substance Use Disorders in the General Population: Results From the National Epidemiologic Survey on Alcohol and Related Conditions. J Clin Psychiatry. 2008 Jul 31;69(7):16576.

46. Mental Health Service Use Among Immigrants in the United States: A Systematic Review | Psychiatric Services [Internet]. [cited 2024 Nov 17]. Available from: https://psychiatryonline.org/doi/10.1176/appi.ps.201500004

47. What Leads Non-U.S.-Born Latinos to Access Mental Health Care?: Social Work in Health Care: Vol 52, No 1 [Internet]. [cited 2024 Nov 17]. Available from: https://www.tandfonline.com/doi/abs/10.1080/00981389.2012.733326

48. Galdas PM, Cheater F, Marshall P. Men and health help-seeking behaviour: literature review. J Adv Nurs. 2005;49(6):616–23.

49. Pattyn E, Verhaeghe M, Bracke P. The gender gap in mental health service use. Soc Psychiatry Psychiatr Epidemiol. 2015 Jul 1;50(7):1089–95.

50. Swetlitz N, Hinton L, Rivera M, Liu M, Fernandez AC, Garcia ME. Barriers and facilitators to depression care among Latino men in a primary care setting: a qualitative study. BMC Prim Care. 2024 Jan 20;25:30.

51. Snowden LR, Hastings JF, Alvidrez J. Overrepresentation of Black Americans in Psychiatric Inpatient Care. Psychiatr Serv. 2009 Jun;60(6):779–85.

52. CDC. Protecting the Nation's Mental Health [Internet]. Mental Health. 2024 [cited 2024 Dec 10]. Available from: https://www.cdc.gov/mental-health/about/what-cdc-is-doing.html

53. Guth M, Artiga S, Published OP. Effects of the ACA Medicaid Expansion on Racial Disparities in Health and Health Care [Internet]. KFF. 2020 [cited 2024 Dec 10]. Available from: https://www.kff.org/medicaid/issue-brief/effects-of-the-aca-medicaid-expansion-on-racial-disparities-in-health-and-health-care/

54. World mental health report: Transforming mental health for all [Internet]. World Health Organization. 2022 [cited 2022 Jul 14]. Available from: https://www.who.int/publications-detail-redirect/9789240049338

55. Mental Health ATLAS 2017 [Internet]. [cited 2022 Jul 16]. Available from: https://www.who.int/publications-detail-redirect/9789241514019

56. Robertson L, Akré ER, Gonzales G. Mental Health Disparities at the Intersections of Gender Identity, Race, and Ethnicity. LGBT Health. 2021 Dec;8(8):526–35.

Figure 1

Click here to access/download;Figure;Figure 1 PLos manuscript.png

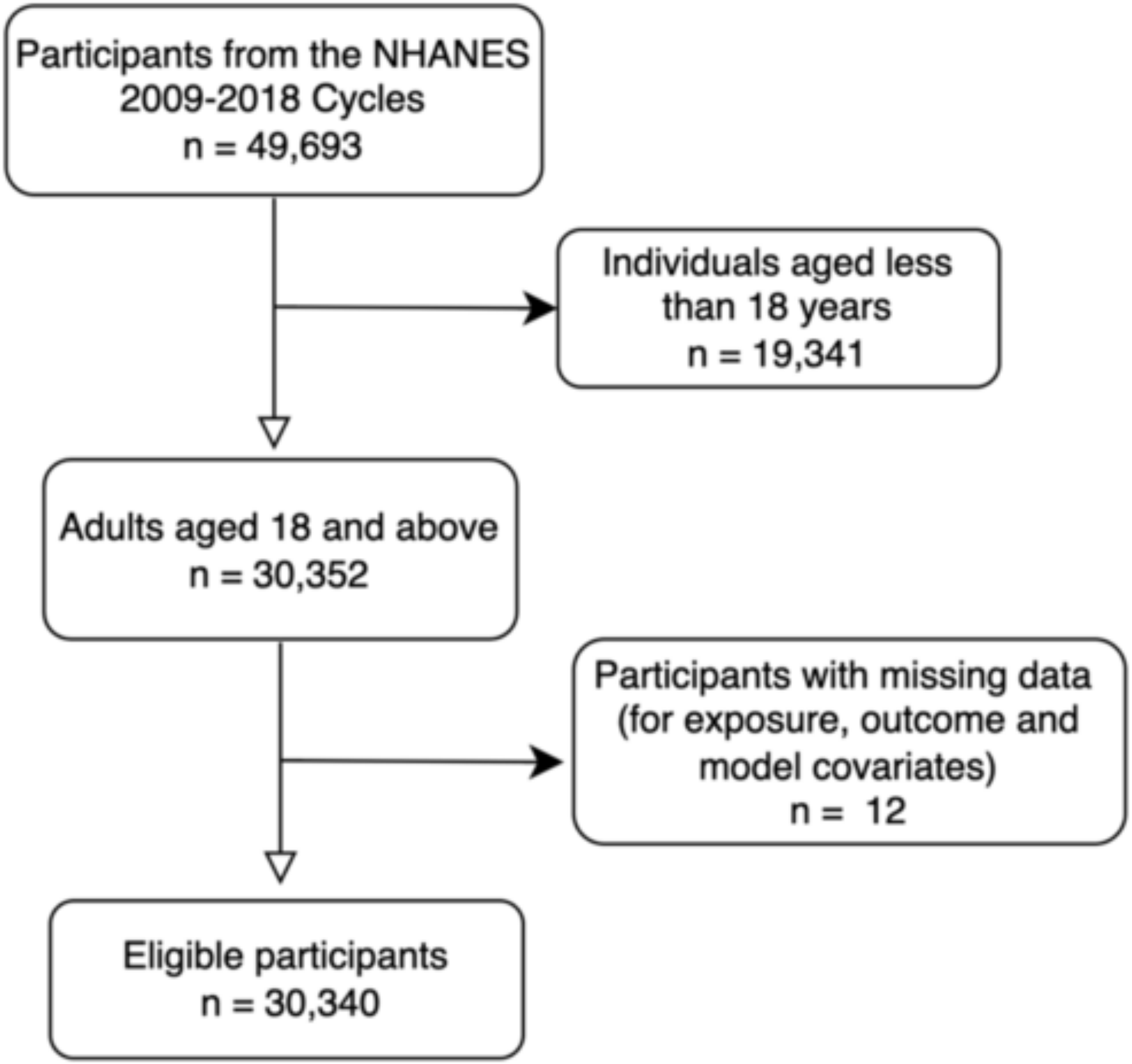

Figure 2

Click here to access/download;Figure;Figure 2 Plos manuscript.png

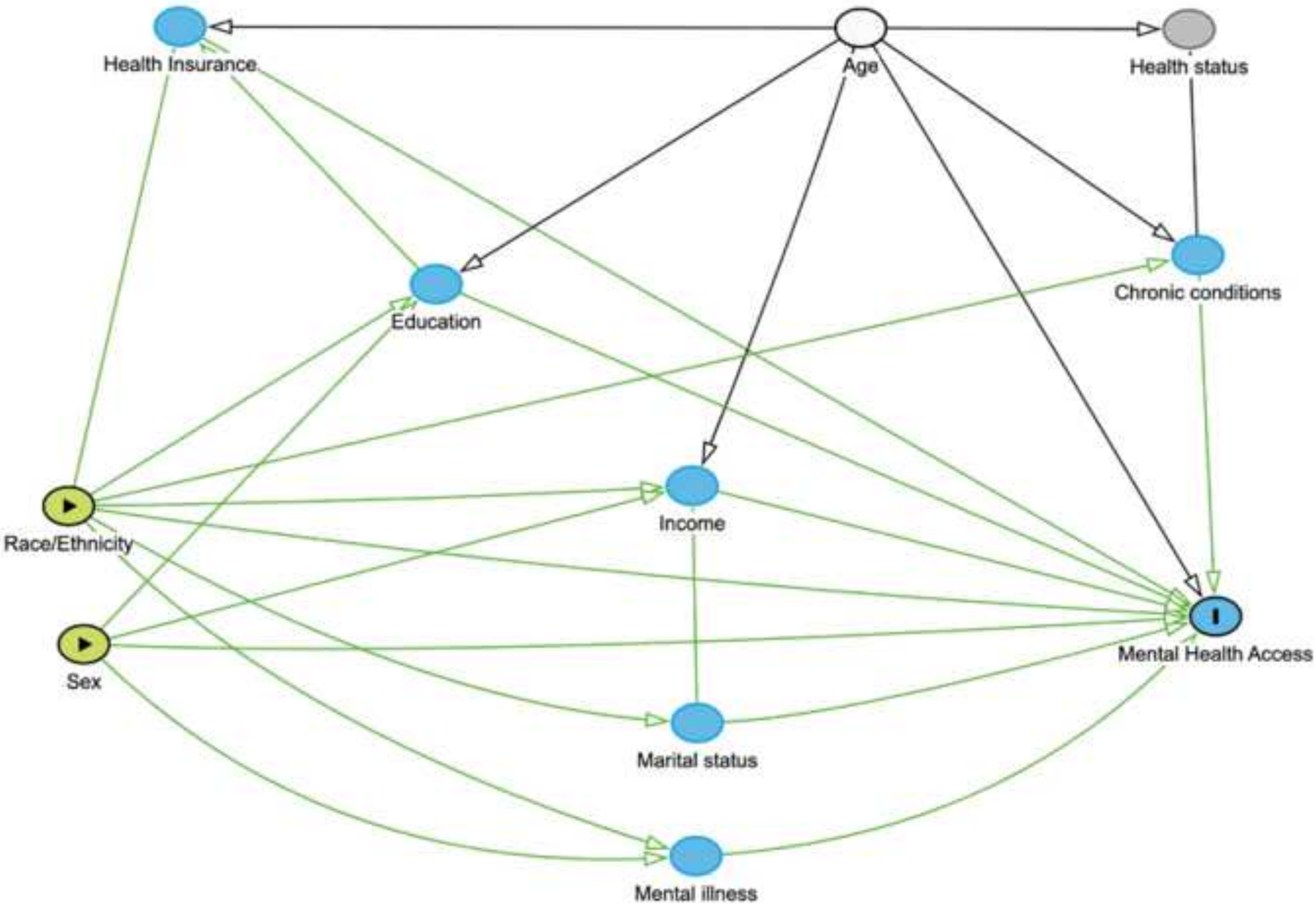

Supplement: Attachment — Submitted filename: PGPH-D-25-01113_reviewer.pdf [file pgph.0004682.s001.pdf]
