# Supplementary material for: Intersectional disparities in mental healthcare utilization by sex and race/ethnicity among US adults: An NHANES study
Source: PLOS Glob Public Health. 2025 Oct 13;5(10):e0004682. doi: 10.1371/journal.pgph.0004682 (PMC12517490; doi:10.1371/journal.pgph.0004682)
Supplement: Attachment — Submitted filename: Intersectionality Manuscript Response to Reviewers.docx [file pgph.0004682.s002.docx]

**Authors' Response to Reviewers**

We thank the reviewers for their insightful and constructive comments, which have improved the quality and clarity of our manuscript. We have addressed each comment point-by-point below, with all changes highlighted in the revised manuscript.

**Reviewer #1**

**Comment 1:** Clarify and define race/ethnicity categories early in the text, and consider commenting on the limitations of these broad categories (especially the heterogeneous “Other” group).

**Response:** We appreciate this suggestion. We have revised the "Exposure" subsection in the Methods to provide a clearer definition of the race/ethnicity categories including the "Non-Hispanic (NH) Other Race" group. Furthermore, as suggested, we have expanded our discussion in the limitations section to more specifically address the heterogeneity of these broad categories, particularly the 'NH Other Race' group, underscoring this as a key limitation of our analysis.

**Comment 2:** Provide greater clarity on limitations of sex vs. gender measures, and the consequences for interpretability, especially in light of intersectionality theory.

**Response:** We thank the reviewer for raising this vital point. We agree that the distinction between sex and gender is a critical consideration for an intersectional study. We have revised the limitations section to provide greater clarity on this issue, explicitly acknowledging that our use of a binary 'sex' variable, due to primary data constraints, is a significant limitation. We have also explained the consequences of this for the interpretability of our findings, particularly in relation to gender-diverse populations.

**Comment 3:** Strengthen the policy/practice recommendations by providing examples of successful interventions or promising approaches for specific subgroups (e.g., Hispanic men).

**Response:** We appreciate this insightful feedback. We agree that providing more specific examples will significantly enhance the practical utility of our manuscript. We have revised the 'Policy and Practice Implications' section to include more concrete and actionable recommendations, such as the integration of services into community organizations and the development of culturally competent programs, to better address the unique disparities identified among key subgroups, including Hispanic men.

**Comment 4:** Conduct a final editorial proofread to catch minor inconsistencies and improve readability.

**Response:** Thank you for this important suggestion. We have thoroughly proofread the manuscript to correct minor inconsistencies and improve overall readability.

**Reviewer #2**

**Comment 1:** Using personal pronouns (like "I" or "we") in the abstract of a research article is not recommended, especially in formal, academic, or scientific writing. Passive voice or third-person constructions are often used. e.g Instead of “We analyzed the data,” write “The data were analyzed.”

**Response:** We appreciate the reviewer’s feedback regarding the use of personal pronouns and the importance of a formal, third-person academic voice. We have revised the abstract and the entire manuscript to remove all personal pronouns and use passive voice or third-person constructions where appropriate, aligning with formal academic writing standards.

**Comment 2:** Two different references style used - please stick to one.

**Response:** We have reviewed and standardized the reference style throughout the manuscript to ensure consistency.

**Comment 3:** Please avoid personal pronouns, this is an academic article. it's not standard practice.

**Response:** As noted in response to Comment 1, we have removed all personal pronouns from the manuscript.

**Comment 4:** Passive voice or third-person constructions should be used.

**Response:** As noted in response to Comment 1, we have implemented passive voice or third-person constructions throughout the manuscript.

**Comment 5:** Please include a table footer to show the significance level. e.g p<0.05. Also an asterisk sign beside a significant value.

**Response:** We have added a table footer to the relevant tables to clearly indicate the significance levels using asterisks, and ensured asterisks are consistently applied to significant values within the table.

**Comment 6:** Please do not use and cite publications over 10 years.

**Response:** We have reviewed our reference list and updated citations to prioritize more recent publications (within the last 10 years) where possible, while retaining essential foundational literature.

**Reviewer #3**

**Comment 1:** Why was the adjusted model limited to age? It says that the other covariates introduced bias when they were adjusted for - but that is not well explained. Furthermore, this is a paper about intersectionality, but excluding overlapping variabilities without more sufficient explanation seems like a mistake both statistically and theoretically. Furthermore, the paper goes on to do sensitivity analyses, but again considers them each individually, which is a weakness of this paper, particularly in light of its focus on intersectionality.

**Response:** We appreciate this important point. Thank you for prompting us to clarify the estimation goal and covariate strategy. We have revised the "Covariate selection" section to provide a more detailed explanation for the adjustment strategy based on the Directed Acyclic Graph (DAG) and to clarify why other covariates were not included in the adjusted models. We also emphasize that the stratified analyses were specifically designed to explore the modifying role of these socioeconomic and health/need-related factors, thereby extending the intersectional analysis beyond just sex and race/ethnicity.

Our primary aim is to estimate the total disparity in mental-healthcare utilization associated with sex and race/ethnicity i.e., disparities that operate through social and structural pathways (e.g., education, income, and insurance), consistent with an intersectionality lens. Per our DAG, those variables lie downstream of the exposures and are therefore potential mediators (and some, potential colliders). Adjusting for them would constitute overadjustment and bias the total disparity toward the null (VanderWeele, 2019). Since age does not sit on the exposure→outcome pathway; we include it as a precision covariate. We have now expanded the DAG explanation to show which variables are treated as mediators or potential colliders.

In summary, to ensure robustness without compromising the estimate, we (i) keep the age-adjusted model as primary; (ii) retain stratified models by education, income, insurance, and depression to examine heterogeneity; and (iii) clarify that p-values in Table 1 may reflect expected compositional differences: because the sample is large and survey-weighted, many between-group differences are likely to be statistically significant. However, these p-values are not used for covariate selection, which is guided by a more robust confounding control strategy (the prespecified DAG) (VanderWeele, 2019).

**Comment 2:** Why is depression the only condition included in the analysis? It may be the only one with severity, but including other conditions would strengthen this. Or to be clear that the topic is depression - not mental health conditions more broadly.

**Response:** We have clarified that our analysis focuses on mental healthcare utilization broadly, and depression was included as a key covariate due to its consistently collected and validated severity measure in NHANES, allowing for a nuanced exploration of its role. Our outcome is any mental-healthcare utilization in the past 12 months, not depression-specific care. We have clarified in the manuscript that depression is used as a proxy for clinical need, not as the focus of the paper. We use PHQ-9 depression severity because NHANES provides a validated, graded measure of mental health need (PHQ-9) suitable for stratification; whereas other psychiatric conditions are not consistently or validly captured across NHANES cycles. We have rephrased parts of the manuscript to ensure that the outcome is consistently referred to as "mental healthcare utilization", while acknowledging depression's role in stratified analysis. We also note the absence of data on other stratifiable mental health conditions as a key limitation of the dataset and analyses.

**Comment 3:** The first sentence of the introduction could be stronger. Did the prevalence change 30 years ago? Is this time period relevant for treatment efforts?

**Response:** Agreed. We have strengthened the opening sentence of the introduction to be more impactful and relevant to the current context of mental health prevalence and treatment efforts. We tightened the opening to foreground the treatment gap and recent U.S. trends, avoiding the ambiguous “30-year” language.

**Comment 4:** The DAG requires more explanation - what do the lines mean and where is that data coming from?

**Response:** We have added a more detailed note to Figure 2 (the DAG) to explain the meaning of the lines and the types of variables represented. We expanded the DAG description to define nodes and arrows, and to clarify which variables are treated as mediators, colliders and independent risk factors of the outcome.

The data for constructing the DAG comes from existing literature and theoretical understanding of the relationships between these variables, which is standard practice for DAGs in epidemiological research (citations already in text). Thus, the DAG reflects assumptions about causal ordering rather than associations estimated from the study data.

**Comment 5:** The Table 1 data is quite extensive and probably too much. I would consider reorganizing or presenting it more clearly. Also, with the p values nearly all below 0.05, this is further evidence for including covariates in the analyses.

**Response:** We have reviewed Table 1 and agree that it is extensive. We have maintained the current structure as it provides a comprehensive overview of the sample characteristics stratified by sex and race/ethnicity, which is important for understanding the baseline demographics of the intersectional groups. However, we have ensured that the discussion of Table 1 in the "Results" section focuses on the most salient findings to avoid overwhelming the reader. Additionally, should the editor or journal staff prefer, we are happy to move the table to the web appendix for improved formatting purposes.

Regarding the p-values, their significance in Table 1 indicates that there are statistically significant differences in these characteristics across sex and race/ethnicity groups, which supports the rationale for an intersectional analysis and the need to consider these factors. As explained in response to Comment 1, our covariate selection for the adjusted models was guided by the DAG to avoid introducing bias, while the stratified analyses explored the modifying effects of these variables. We also note that NHANES’ large, weighted sample makes most between-group tests significant; however, baseline p-values are not a criterion for covariate control in a DAG-guided analysis.

**Comment 6:** The discussion needs a lot of work. Both to community the findings more clearly, but also the related literature is not well organized and sometimes problematic. The results should be presented similarly (eg, 41% less likely vs 0.75-0.81), but there is far too much of restating the results and the linkages to related literature is weak. For example, the paragraph - "several factors might contribute to these observed.... has the quality of care from providers, stigma, and structural barriers. These are massive topics and then to jump to potential explanations of why Hispanic males might have lower access tried to cover too much space. I would recommend revising.

**Response:** Thank you for raising this important point. We acknowledge that the discussion section required significant refinement. We have thoroughly revised the "Discussion" section to improve clarity, organization, and the integration of findings with existing literature. Key changes include:

- We have refined the presentation of results in the discussion, focusing on interpretation and implications rather than simply restating numerical findings. We have also ensured consistency in how percentages and prevalence ratios are presented.
- We have strengthened the connections between our findings and the existing literature, ensuring that citations are relevant and directly support the points being made.
- We have restructured paragraphs to provide more focused and coherent explanations for observed disparities, avoiding broad generalizations.
- We have worked on improving the overall flow and cohesion of the discussion, ensuring that each paragraph builds logically on the previous one.

**Comment 7:** Building on the challenges of the discussion, the policy and practice implications lack focus and aren't meaningfully related to the findings of this paper.

**Response:** Thank you for raising this crucial point which we agree with. We have significantly revised the "Policy and Practice Implications" section to ensure it is directly linked to our findings and offers more focused implications recommendations.

**Comment 8:** Limitations: This is the first mention of LGBTQ+ people, so it seems a bit too much on this and does not reflect the other limitations of this paper (including that intersectionality is more than race and gender. Or that race is a social construct. Using a huge data set. Lack of data on other mental health conditions. Etc.).

**Response:** We agree that the limitations section needed to be more comprehensive and balanced. We have revised this section to:

- Ensure that the mention of LGBTQ+ populations flows logically from the discussion of sex/gender limitations and the broader concept of intersectionality.
- Explicitly stated that intersectionality extends beyond sex and race/ethnicity, acknowledging other important axes of identity.
- Acknowledge the implication of lack of data on other mental conditions.
- While addressing limitations, briefly reiterate the strengths of the study, such as the use of a large nationally representative sample and an intersectional approach.

**Reviewer #4**

**Comment 1:** However, ” culturally informed strategies and wider structural interventions...(Abstract & conclusion)” is beautiful but outside of data. What informs this conclusion? It seems MH services are available, but the actual uptake is affected by the sociodemographic characteristics. Also, there could be other systemic factors, like a lack of task-shifted services, that are affecting access and utilization of the services, not necessarily culturally informed strategies.

**Response:** We appreciate this feedback and agree that the link between our findings and the concluding recommendations needed to be more explicit. We have revised the "Discussion" and "Conclusion" sections to better articulate how our data, particularly the stratified analyses, inform the need for culturally informed strategies and structural interventions. We agree that our dataset does not directly evaluate culturally informed interventions, and we have reframed these points accordingly. We have rephrased to ensure the recommendations are clearly rooted in the study's findings.

**Comment 2:** ‘Social determinants of health’, a close concept to socio-demographics, is mentioned in the introduction and discussion. Adding a sentence or two explaining distinct causative and impactful differences in mental health utilization would help provide more clarity in their contributions.

**Response:** Thank you for this point. We have clarified the relationship between social determinants of health and sociodemographic factors, and their distinct contributions to mental health utilization.

**Comment 3:** Recommendation: Consider the ‘culturally informed strategies and structural interventions’ as a recommendation for further study.

**Response:** Thank you for this suggestion. We have reflected this in the policy and practice implication section.

**Comment 4:** Limitation: Please add why this article can not be generalized to the LMICs or provide a brief relevance of this article to the LMICs in the discussion.

**Response:** We have added a statement in the "Limitations" section to address the generalizability of our findings outside the United States including to Low- and Middle-Income Countries (LMICs).
